# RETRACTION: MiRNA‐545 Negatively Regulates the Oncogenic Activity of EMS1 in Gastric Cancer

**DOI:** 10.1002/cam4.71011

**Published:** 2025-06-30

## Abstract

**RETRACTION**: MaM. , ZhaoJ. , WuQ. , XiaoK. , LiS. , ZhuH. , LiuC. , XieH. , and ZuoC. , “MiRNA‐545 Negatively Regulates the Oncogenic Activity of EMS1 in Gastric Cancer,” Cancer Medicine 7, no. 6 (2018): 2452–2462, 10.1002/cam4.1520.29733519 PMC6010719

The above article, published online on 07 May 2018 in Wiley Online Library (wileyonlinelibrary.com), has been retracted by agreement between the authors; the journal Editor‐in‐Chief, Stephen Tait; and John Wiley & Sons Ltd.

The retraction has been agreed due to the use of contaminated cell lines in this article. Both the cell lines used to demonstrate the regulatory role of miR‐545 in EMS1 expression (SGC‐7901 & BGC‐823) have been reported to be contaminated with HeLa [1, 2], as has cell line MGC‐803 [3, 4]. Thus, the editors consider the conclusions of this article to be invalid.

We did not find any evidence of misconduct by the authors.

**References:**

[1] 
YeF.
, 
ChenC.
, 
QinJ.
, 
LiuJ.
, and 
ZhengC.
, “Genetic Profiling Reveals an Alarming Rate of Cross‐Contamination Among Human Cell Lines Used in China,” The FASEB Journal
29, no. 10 (2015): 4268–4272, 10.1096/fj.14-266718.26116706

[2] 
BianX.
, 
YangZ.
, 
FengH.
, 
SunH.
, and 
LiuY.
, “A Combination of Species Identification and STR Profiling Identifies Cross‐contaminated Cells from 482 Human Tumor Cell Lines,” Scientific Reports
7 (2017): 9774, 10.1038/s41598-017-09660-w.28851942
PMC5575032

[3] 
CaoF.
, 
SunH.
, 
YangZ.
, 
BaiY.
, 
HuX.
, 
HouY.
, 
BianX.
, and 
LiuY.
, “Multiple Approaches Revealed MGc80‐3 as a Somatic Hybrid with HeLa Cells Rather than a Gastric Cancer Cell Line,” International Journal of Cancer
154, no. 1 (2024): 155–168, 10.1002/ijc.34677.37543987

[4] 
YangM.
, 
HeJ.
, 
XiaS.
, 
WangY.
, 
XiongJ.
, 
LiaoC.
, 
LiN.
, 
QuS.
, and 
ShenC.
, "Investigation of the Mixed Origins of the MGC‐803 Cell Line Reveals That It Is a Hybrid Cell Line Derived from HeLa," Human Cell
37 (2024): 560–566, 10.1007/s13577-023-01011-4.38079103

**RETRACTION**: M. Ma , J. Zhao , Q. Wu , K. Xiao , S. Li , H. Zhu , C. Liu , H. Xie , and C. Zuo , “MiRNA‐545 Negatively Regulates the Oncogenic Activity of EMS1 in Gastric Cancer,” Cancer Medicine 7, no. 6 (2018): 2452–2462, 10.1002/cam4.1520.29733519 PMC6010719

The above article, published online on 07 May 2018 in Wiley Online Library (wileyonlinelibrary.com), has been retracted by agreement between the authors; the journal Editor‐in‐Chief, Stephen Tait; and John Wiley & Sons Ltd.

The retraction has been agreed due to the use of contaminated cell lines in this article. Both the cell lines used to demonstrate the regulatory role of miR‐545 in EMS1 expression (SGC‐7901 & BGC‐823) have been reported to be contaminated with HeLa [1, 2], as has cell line MGC‐803 [3, 4]. Thus, the editors consider the conclusions of this article to be invalid.

We did not find any evidence of misconduct by the authors.
